# Compositional and optical properties of SiO_*x*_ films and (SiO_*x*_/SiO_*y*_) junctions deposited by HFCVD

**DOI:** 10.1186/1556-276X-9-422

**Published:** 2014-08-21

**Authors:** Diana E Vázquez-Valerdi, Jose A Luna-López, Jesús Carrillo-López, Godofredo García-Salgado, Alfredo Benítez-Lara, Néstor D Espinosa-Torres

**Affiliations:** 1 IC-CIDS Benemérita Universidad Autónoma de Puebla, Ed. 103 C o D, Col. San Manuel, C.P, Puebla, Pue 72570, Mexico

**Keywords:** HFCVD, PL, FTIR, Transmittance, Thermal annealing, Si-nc

## Abstract

In this work, non-stoichiometric silicon oxide (SiO_*x*_) films and (SiO_*x*_/SiO_*y*_) junctions, as-grown and after further annealing, are characterized by different techniques. The SiO_*x*_ films and (SiO_*x*_/SiO_*y*_) junctions are obtained by hot filament chemical vapor deposition technique in the range of temperatures from 900°C to 1,150°C. Transmittance spectra of the SiO_*x*_ films showed a wavelength shift of the absorption edge thus indicating an increase in the optical energy band gap, when the growth temperature decreases; a similar behavior is observed in the (SiO_*x*_/SiO_*y*_) structures, which in turn indicates a decrease in the Si excess, as Fourier transform infrared spectroscopy (FTIR) reveals, so that, the film and junction composition changes with the growth temperature. The analysis of the photoluminescence (PL) results using the quantum confinement model suggests the presence of silicon nanocrystal (Si-nc) embedded in a SiO_*x*_ matrix. For the case of the as-grown SiO_*x*_ films, the absorption and emission properties are correlated with quantum effects in Si-nc and defects. For the case of the as-grown (SiO_*x*_/SiO_*y*_) junctions, only the emission mechanism related to some kinds of defects was considered, but silicon nanocrystal embedded in a SiO_*x*_ matrix is present. After thermal annealing, a phase separation into Si and SiO_2_ occurs, as the FTIR spectra illustrates, which has repercussions in the absorption and emission properties of the films and junctions, as shown by the change in the A and B band positions on the PL spectra. These results lead to good possibilities for proposed novel applications in optoelectronic devices.

**PACS:**

61.05.-a; 68.37.Og; 61.05.cp; 78.55.-m; 68.37.Ps; 81.15.Gh

## Background

Silicon is the semiconductor material predominant in the microelectronics industry. However, it has been long considered unsuitable material for optoelectronic applications [1], due to its indirect band gap, which means it is a poor light emitter. Though, after discovery of visible light emission at room temperature in the porous silicon by Canham [2] in 1990, many investigators have studied emission properties of materials with silicon compounds as the non-stoichiometric silicon oxide (SiO_*x*_), which has gained increasing interest in the research community due to the formation of silicon nanocrystals embedded in the matrix, implying low-dimensional effects and thus determines an efficient emission of visible light, even at room temperature [3,4]. In the SiO_*x*_ films, the absorption and emission properties are correlated with quantum effects in silicon nanoparticles, and also associated with defects [5]. From the technological standpoint, the average size of a silicon nanoparticle (Si-np) offers band gap widths, which opens the possibility to tune the emission of light using nanostructured thin films in novel optoelectronic devices. The goal of this work is to study and investigate the compositional and optical properties of SiO_*x*_ films and (SiO_*x*_/SiO_*y*_) junctions obtained by hot filament chemical vapor deposition (HFCVD) technique, as-grown and after a further annealing, in order to have a broad and solid view of the behavior of the material by varying the growth temperature and mainly its thickness, which opens the possibility for proposed novel applications in a future work.

## Methods

SiO_*x*_ films and (SiO_*x*_/SiO_*y*_) junctions were obtained by HFCVD technique in the range of temperatures from 900°C to 1,150°C and deposited on quartz and n-type silicon (100) substrates with 1 to 10 Ω cm resistivity, using quartz and porous silicon as the reactive sources. The deposition time was 5 min for the SiO_*x*_ films and of 10 min for the (SiO_*x*_/SiO_*y*_) junctions due to that the time for each film of the junction was of 5 min. The chemical reaction for the grown non-stoichiometric silicon oxide in the HFCVD technique is pictured in [6,7]. The relationship between the filament temperature (approximately 2,000°C) and the variation of the source-substrate distance (dss) of 4, 5, and 6 mm provides a change in the growth temperature (Tg) of 1,150°C, 1,020°C, and 900°C, respectively, which was measured with a thermocouple in the reaction zone. The SiO_*x*_ films were deposited at 1,150°C, 1,020°C, and 900°C. Three samples of each kind have been studied. The (SiO_*x*_/SiO_*y*_) junctions were made with two films deposited at two different temperatures, obtaining six possible combinations, 1,150°C/1,020°C, 1,150°C/900°C, 1,020°C/1,150°C, 1,020°C/900°C, 900°C/1,150°C, and 900°C/1,020°C. Three samples of each kind have been studied. The changes in the dss and Tg, consequently, modify the silicon excess and defects in the non-stoichiometric SiO_*x*_ films. The substrates were carefully cleaned with a metal oxide semiconductor standard cleaning process and the native oxide was removed with an HF buffer solution before being introduced into the reactor. The thermal annealing was made using a nitrogen atmosphere at 1,100°C for 1 h. Several spectroscopic characterization techniques were used. The film thickness was measured using a Dektak 150 profilometer (Veeco Instruments Inc., Plainview, NY, USA). Room-temperature transmittance of the SiO_*x*_ films was measured using a UV–Vis-NIR Cary 5,000 system (Agilent Technologies Inc., Santa Clara, CA, USA). The transmittance signal was collected from 200 to 1,000 nm with a resolution of 0.5 nm. FTIR spectroscopy measurements were done using a Bruker system model vector 22 (Bruker Instruments, Bellirica, MA, USA). Photoluminescence (PL) response was measured at room temperature using a Horiba Jobin Yvon spectrometer model FluroMax 3 (Edison, NJ, USA) with a pulsed xenon source whose detector has a multiplier tube, which is controlled by a computer. The samples were excited using a 250-nm radiation, and the PL response was recorded between 380 and 1,000 nm with a resolution of 1 nm.

## Results and discussion

**Figure 1:**
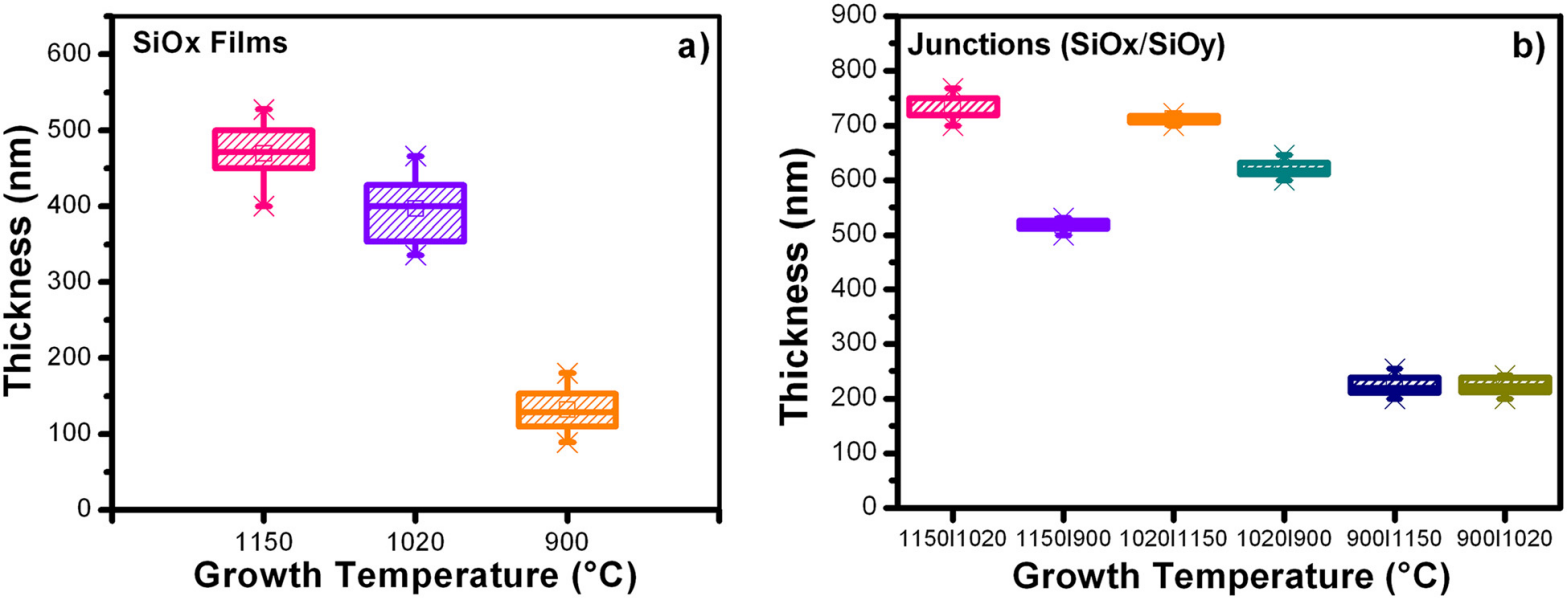
**Thickness as function of Tg of the SiO**_***x ***_**films (a) and (SiO**_**x**_**/SiO**_**y**_**) junctions (b).**

The thicknesses of the SiO_*x*_ films and (SiO_*x*_/SiO_*y*_) junctions as a function of the growth temperature (Tg) are shown in Figure 1 as box statistic charts, where the mean, maximum, and minimum values are shown, the width of the box denotes the standard deviation of the measurements. The Tg and the deposition time affect the thickness of the SiO_*x*_ films and (SiO_*x*_/SiO_*y*_) junctions. When Tg was decreased and the deposition time was less, thinner samples and a more uniform deposition were obtained due to lesser amount of volatile precursors likely to deposit. Figure 1b shows that the junctions have a better uniformity than the films (Figure 1a) because the standard deviation (width of the box) is less. This could be due to a possible ‘annealing’ of the first SiO_*x*_ film deposited. In this case, the first layer has received annealing twice, which would certainly lead to a different formation. Then, these results show that the double annealing procedure and the order of layers have a very strong impact on the thickness. The tendency is that the more annealing, the thicker the layer. For the SiO_*x*_ films, average thicknesses were obtained of 500 to 100 nm and for the (SiO_*x*_/SiO_*y*_) junctions of 750 to 200 nm.

**Figure 2:**
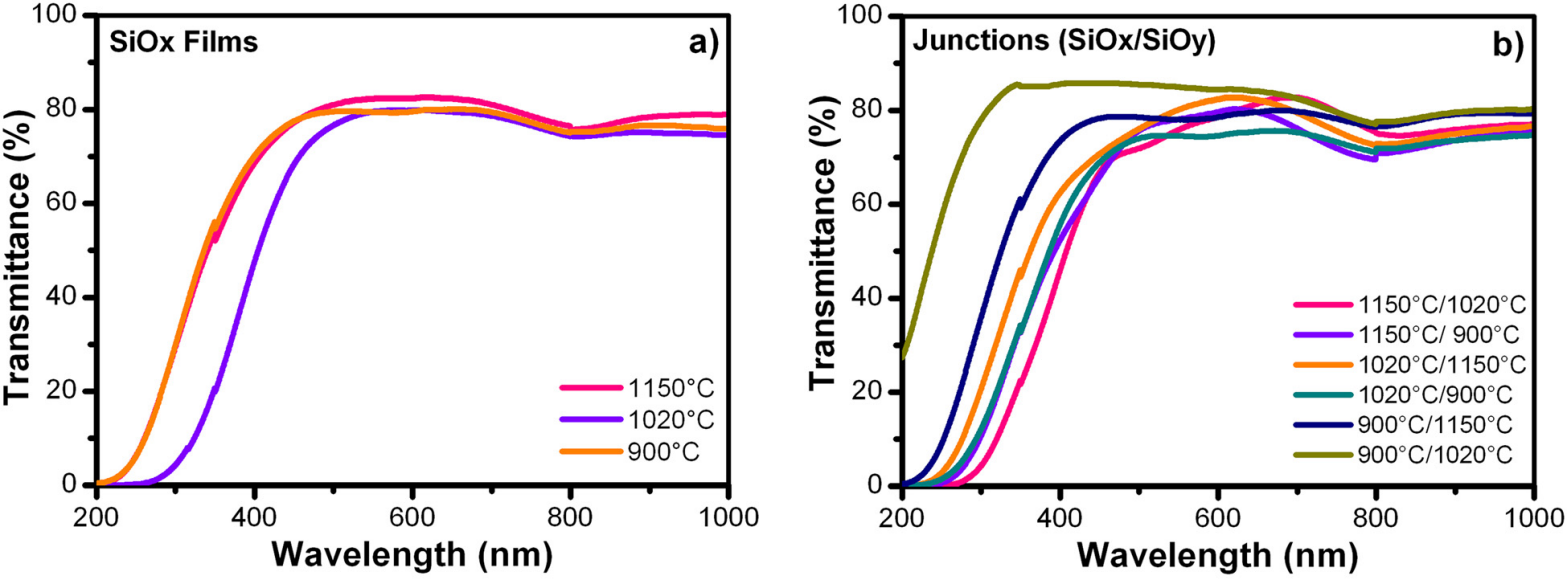
**Transmittance spectra as function of Tg of the SiO**_***x ***_**films (a) and (SiO**_***x***_**/SiO**_***y***_**) junctions (b).**

Figure 2 shows the UV–Vis transmittance spectra of SiO_*x*_ films and (SiO_*x*_/SiO_*y*_) junctions as-grown deposited on quartz. All the samples exhibited a relatively high transmittance (>70%) between 600 and 1,000 nm. The change in the growth temperature produces a change in the stoichiometry of the SiO_*x*_ films and thickness, which produces a shift of the absorption edge. In a previous work, where the SiO_*x*_ films were characterized by XEDS [7], the information on change in stoichiometry of SiO_*x*_ were reported; where *x* for the film deposited at 1,150°C was 1.27, at 1,020°C was 1.81, and at 900°C was 1.66; these values are approximates. Figure 2a shows a similar absorption edge for 900°C and 1,150°C, while 1,020°C is shifted; therefore, a random result is obtained. Figure 2b shows that there is an extremely large difference between junctions; this seems to imply that the double annealing procedure and the order of layers have a very strong impact on optical properties. The overall trend seems as the more annealing is received, the less transparent the sample is in visible range - i.e., annealing is facilitating formation of absorbing/emitting states (defects or NCs) [8].

**Figure 3:**
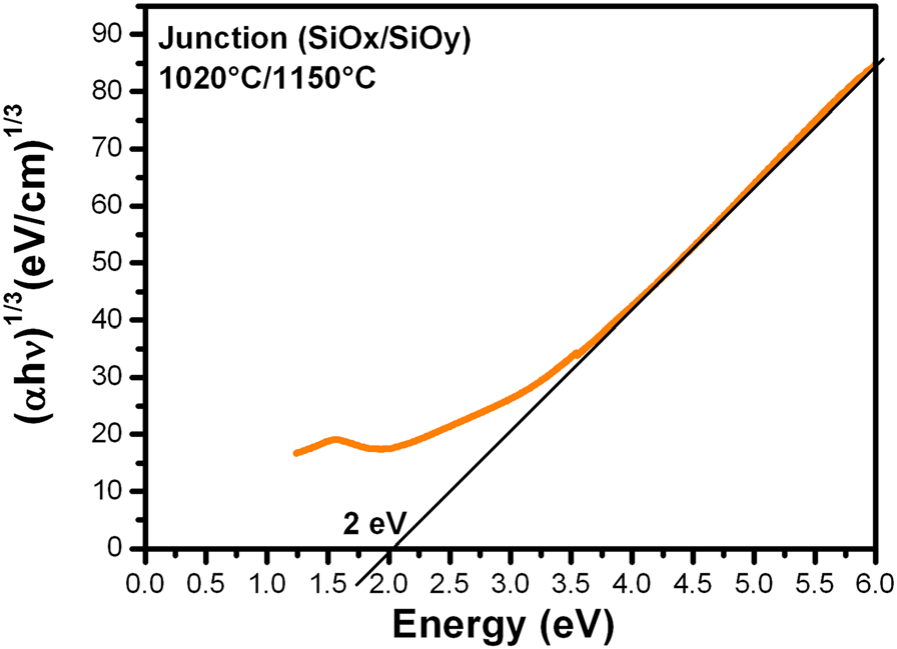
**(αhν)**^**1/3**^**versus energy (hν).** Example to obtain the approximate value of Eg by the relationship known as Tauc plot.

The approximate values of the energy band gap (Eg) were obtained by the relationship known as Tauc plot [9-11] as shown in Figure 3. The methodology for obtaining these approximate values of Eg has been described in a previous work [7], where the absorption coefficients α(λ) were determined from transmission spectra and was obtained in the order of 10^3^ to 10^4^ cm^-1^. The Eg decreases as the growth temperature increases; the optical energy band gap of the SiO_*x*_ films is in the range of energies of 2.15 to 1.8 eV and of the (SiO_*x*_/SiO_*y*_) junctions is in the range of energies of 2.25 to 1.8 eV. When *x* decreases from 2.0 in a-SiO_*x*_, the valence band edge moves up, as the increased Si-Si bond states are gradually overlapped with the oxygen nonbonding states (ONS) and finally spread out into the Si valence band. Simultaneously, the conduction band edge also moves down. The final result is that the optical band gap decreases nonlinearly when Si concentration continually increases [5]. Therefore, we may assume that the Si excess increases as the growth temperature increases.

**Figure 4:**
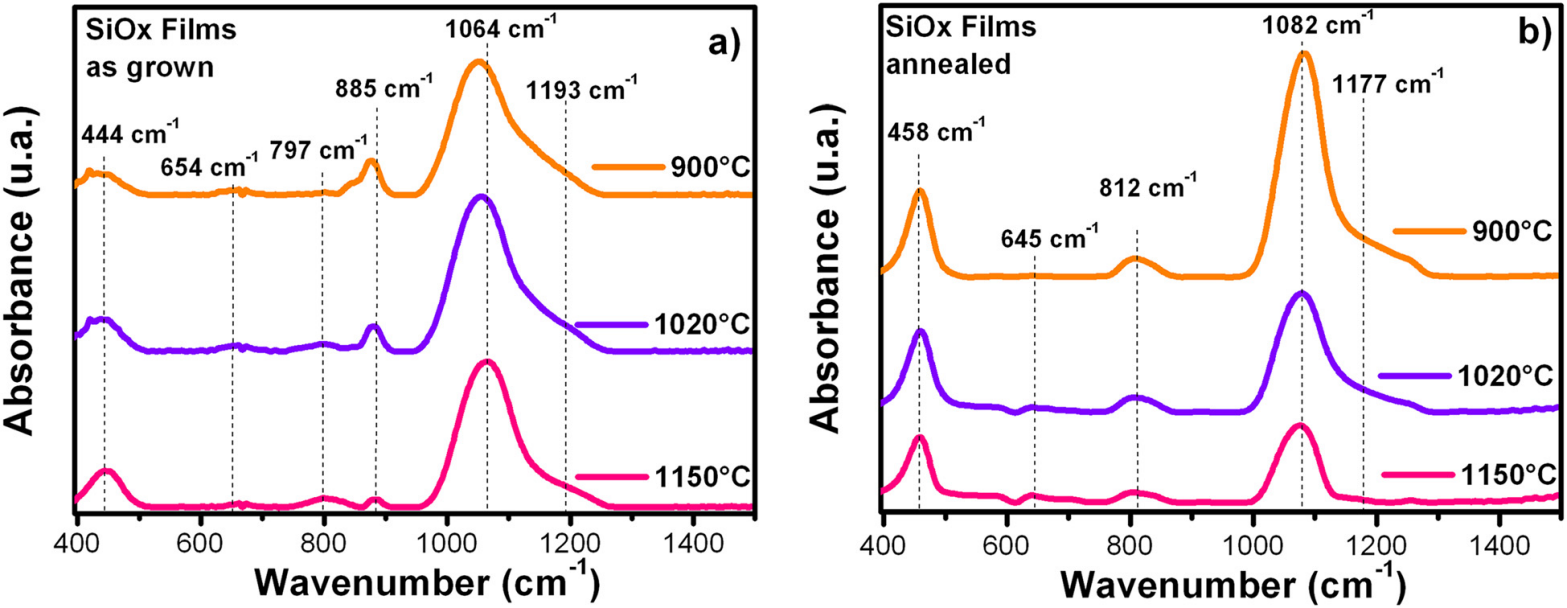
**FTIR spectra from SiO**_***x***_**films as-grown (a) and after further annealing (b).**

**Figure 5:**
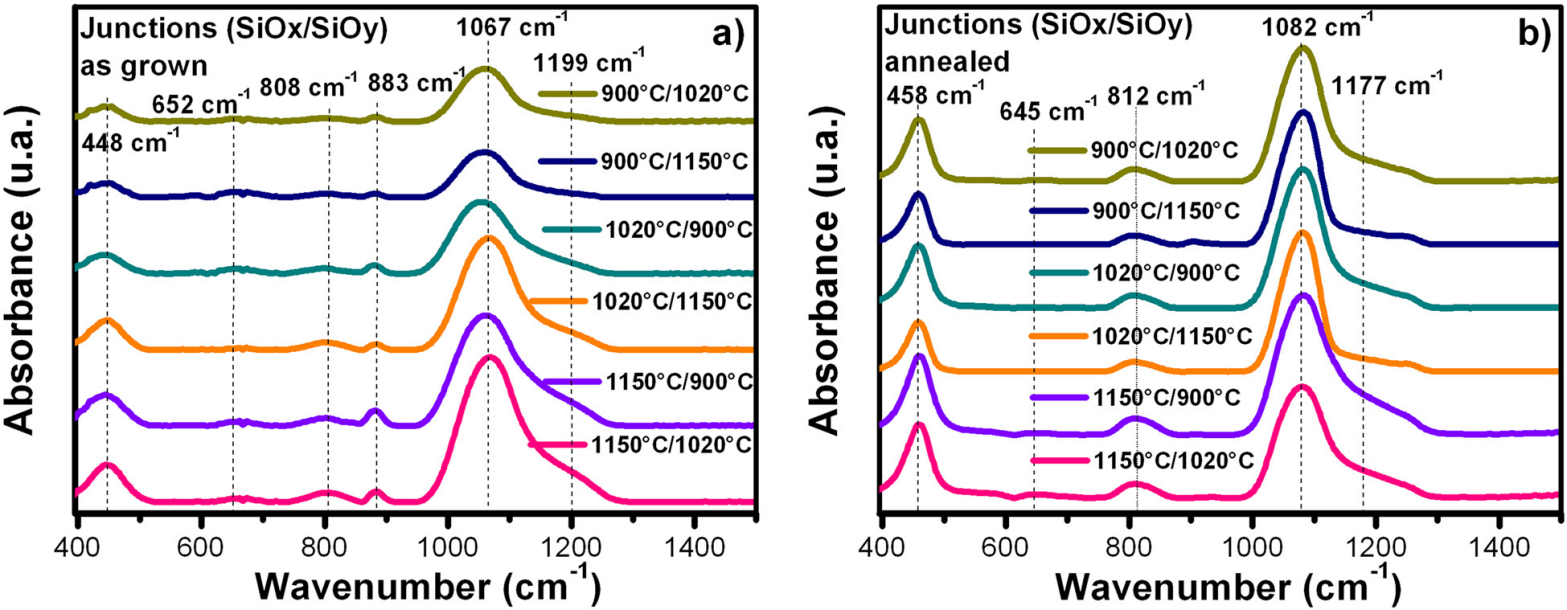
**FTIR spectra from (SiO**_***x***_**/SiO**_***y***_**) junctions as-grown (a) and after further annealing (b).**

Figures 4 and 5 show the FTIR spectra from SiO_*x*_ films and (SiO_*x*_/SiO_*y*_) junctions as-grown and after a further annealing, respectively. The IR vibration bands are listed in Table 1 for the SiO_*x*_ films and in Table 2 for the (SiO_*x*_/SiO_*y*_) junctions. All the spectra show the absorption peaks characteristic of SiO_2_, which correspond to the vibration modes of stretching (1,082 cm^-1^), bending (812 cm^-1^), and rocking (458 cm^-1^) of Si-O-Si [12,13]. Before thermal annealing, these peaks show a shift to lower wavenumber with decreasing of the growth temperature (Tg), indicating the change in the silicon excess. After thermal annealing, these shifts disappear and the position of the vibration modes corresponding to SiO_2_, thus indicating a phase separation. Before thermal annealing, a vibration band approximately at 885 cm^-1^, assigned to Si-H bending and Si-O of Si-O-H [14-16], appeared with a shoulder at 810 cm^-1^ related to the Si-O bending vibration band. When the Tg was higher, the intensity of the Si-O bending vibration band increased and shift at the same time that the peak at 870 cm^-1^ decreased, and the Si-O bending vibration band became more apparent. These bonds are present in the films due to the incorporation of hydrogen in the experimental process, but after the films were thermally annealed, the band at 885 cm^-1^, disappeared due to the desorption of hydrogen to high temperature and the characteristic absorption peaks of SiO_2_ were more noticeable. The Si-O stretching vibration band shifted to higher frequencies after thermal annealing indicating a phase separation and its width reduced due to an increment in the oxygen concentration. Also, all samples exhibit a peak around 645 to 664 cm^-1^, which has been associated with Si-H wagging bonds or neutral oxygen vacancies [17]. These bonds are almost imperceptibly present in both cases.

**Table 1:** **IR vibration bands**[12-17]**of the SiO**_***x ***_**films before and after thermal annealing**

**Vibration mode**	**Peak position (cm**^**-1**^**)**					
	**As grown**			**Annealed**		
	**1,150°C**	**1,020°C**	**900°C**	**1,150°C**	**1,020°C**	**900°C**
Si-O rocking	444	436	429	458	458	458
Si-O bending	797	800	810	812	812	812
Si-O stretching	1,064	1,055	1,048	1,082	1,082	1,082
Si-H wagging	654	649	645	645	645	-
Si-H bending	885	879	875	-	-	-

**Table 2:** **IR vibration bands**[12-17]**of the (SiO**_***x***_**/SiO**_***y***_**) junctions before and after thermal annealing**

	**Vibration mode**	**Peak position (cm**^-**1**^**)**					
		**1,150°C/1020°C**	**1,150°C/900°C**	**1,020°C/1,150°C**	**1,020°C/900°C**	**900°C/1,150°C**	**900°C/1,020°C**
**As grown**	Si-O rocking	447	444	442	442	440	440
	Si-O bending	808	803	800	800	797	796
	Si-O stretching	1,067	1,063	1,063	1,055	1,055	1,054
	Si-H wagging	664	659	645	-	652	651
	Si-H bending	883	883	880	879	879	877
**Annealed**	Si-O rocking	458	458	458	458	458	458
	Si-O bending	812	812	812	812	812	812
	Si-O stretching	1,082	1,082	1,082	1,082	1,082	1,082
	Si-H wagging	645	640	-	637	-	-
	Si-H bending	-	-	-	-	-	-

**Figure 6:**
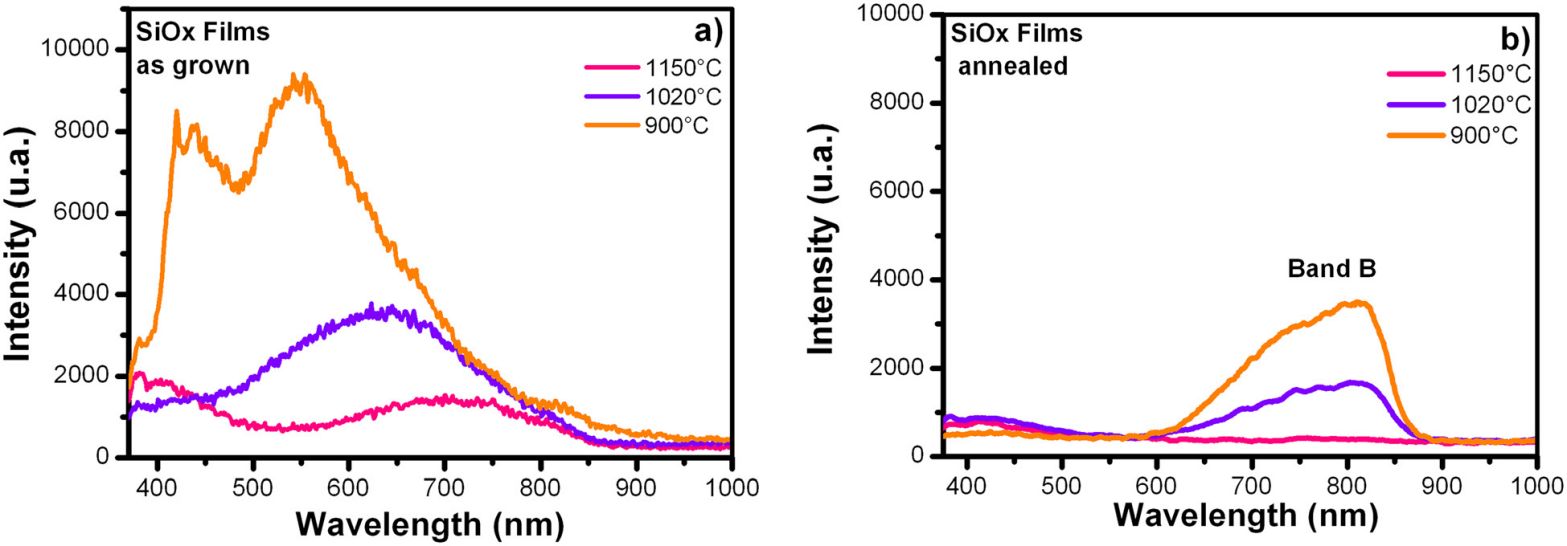
**PL spectra from SiO**_***x***_**films as-grown (a) and after further annealing (b).**

**Figure 7:**
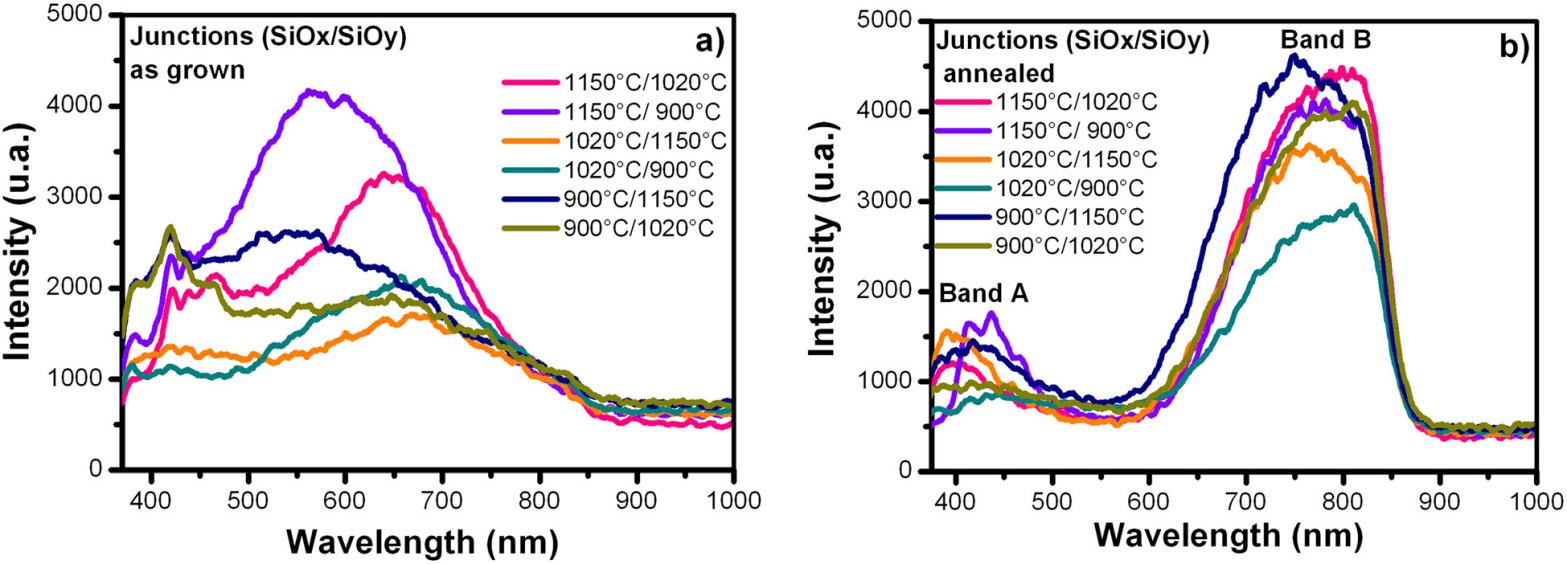
**PL spectra from (SiO**_***x***_**/SiO**_***y***_**) junctions as-grown (a) and after further annealing (b).**

**Figure 8:**
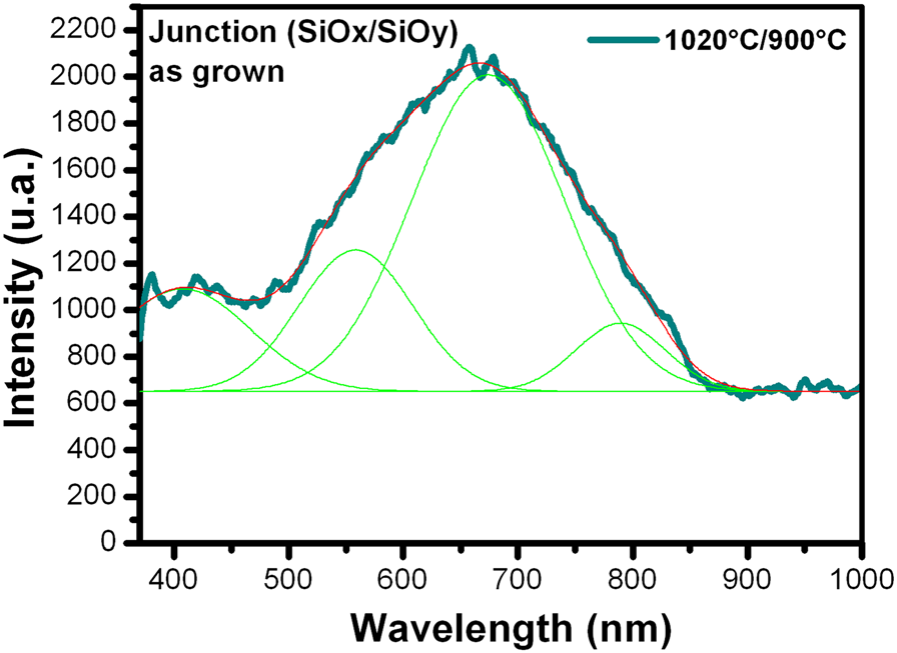
Example of the deconvolution applied to PL spectra.

Figures 6 and 7 show the PL spectra from SiO_*x*_ films and (SiO_*x*_/SiO_*y*_) junctions as-grown and after further annealing, respectively.A wide PL spectrum with a similar shape to a Gaussian curve is observed for all the samples. For this reason, the deconvolution was applied to the PL spectra, with which several peaks were obtained as shown in Figure 8; each of the peaks represent the emission individually, which are the components of the PL spectra. Therefore, each peak has different origins.

In general, in the PL spectra of the SiO_*x*_ films and (SiO_*x*_/SiO_*y*_) junctions as-grown, there are several emission bands covering a wide spectral range from 380 to 850 nm (violet-near infrared range). After further annealing, the PL spectra there are two main bands, band A of 380 to 495 nm (violet-blue range) with a relatively weak PL intensity and band B of 590 to 875 nm (orange-near infrared range). In this case, the green-yellow band disappears. The PL spectra of the SiO_*x*_ films, as-grown (see Figure 6a) exhibit a higher PL intensity with respect to the PL intensity after further annealing (see Figure 6b). The PL spectra of (SiO_*x*_/SiO_*y*_) junctions as-grown (see Figure 7a) illustrate a relatively weak PL intensity with respect to PL intensity after further annealing (see Figure 7b). The origin of the PL emission is still object of debate; two models have been proposed. The first model relates the PL to quantum confinement effects (QCE) [18,19] in Si-nc's. The second model attributes the PL to defects in the matrix associated with oxygen vacancies or defects in the interface SiO_2_/Si-nc [16,19]. Figure 6a shows that the SiO_*x*_ films, as-grown, have a maximum emission peak that suffers a blue shift and an increase in the intensity as the growth temperature decreases. This behavior could be related to the presence of Si-nc's as was observed in a previous work, where the SiO_*x*_ films were characterized by HRTEM [7], and the average size of Si-nc was obtained, which reduced along with the decreasing growth temperature. Therefore, in this case, the PL spectra are analyzed in terms of a quantum confinement model [18,19]:

$$\lambda \left(\mathrm{nm}\right)=\frac{1.24\;\mathrm{\mu m}}{E_{N}\left(\mathrm{eV}\right)}\;=\frac{1.24\;\mathrm{\mu m}}{\left[1.12+\left(3.73/d^{1.39}\right)\right]}$$

It corresponds to the radiative recombination of electron–hole pairs in the Si-nc, where *d* and *E*_*N*_ are the diameter and energy of the Si-nc, respectively, and *λ*(nm) is the wavelength of the Si-nc emission. Table 3 shows the theoretical values of average sizes of Si-nc calculated from the main peak of the PL spectra, where the size of the Si-nc reduces and the energy band gap increases with decreasing the growth temperature. Table 3 also shows the values of average sizes of Si-nc obtained by HRTEM [7]. So the blue shift of PL with decreasing Si concentration and growth temperature, i.e., decreasing Si-nc size, is in good agreement with the QCE. Besides, in these SiO_*x*_ films, the absorption and emission are correlated with quantum effects in Si-nc because the theoretical values of *E*_*N*_ are similar to the approximate values of the optical band gap (Eg) that were obtained by the relationship known as Tauc plot (see Figure 3).

**Table 3:** Theoretical and experimental values

**Tg (°C)**	**Gap *E***_***N ***_**of Si-nc (eV)**	**Diameter of Si-nc (nm)**	**Diameter of Si-nc (nm) by HRTEM [**[7]**]**
1,150	1.67	3.96	5.5
1,020	1.89	3.11	4
900	2.28	2.31	2.5

Values of the *E*_*N*_ and the diameter of Si-nc calculated from the PL spectra of the SiO_*x*_ films as-grown as a function of the Tg.

However, PL spectra are very wide, which indicates that two possible mechanisms are involved. Then, the second mechanism of light emission that was considered in the SiO_*x*_ films is related to some kinds of defects produced during the growth process, as shown in the FTIR spectra, such as, weak oxygen bonds (WOB), neutral oxygen vacancy (NOV), non-bridging oxygen hole center (NBOHC), positively charged oxygen vacancy (E' center), interstitial oxygen molecules, and peroxide radicals [19-24]. Some of these defects, such as NOV and NBOHC are the principal radiative recombination centers or the luminescence centers. So, the different peaks that were defined by the deconvolution of the PL spectra are related to different kinds of defects, as listed in Table 4.

**Table 4:** **Defect types**[19-24]**linked with the peak position**

**Defect type**	**Peak position (nm)**		
	**As grown**		
	**1,150°C**	**1,020°C**	**900°C**
WOB	400	410	
NOV defect			436
E'δ defect	-	540	543
NBOHC	-	-	658
None identified	739	758	-

Position obtained by deconvolution from PL spectra of the SiO_*x*_ films as function of the Tg.

Figure 7a shows that the PL spectra of the (SiO_*x*_/SiO_*y*_) junctions, as-grown, emit in a wide spectral range. This is due to the junction of two films with different properties and stoichiometries, so these spectra have a behavior mixed with respect to the SiO_*x*_ films (see Figure 6a), where again the first film deposited dominates the behavior of the junction. For this case, the PL spectra are not analyzed in terms of a quantum confinement model, because the PL spectra have not a maximum emission peak that suffers a blue shift. So only the second mechanism of light emission was considered in the (SiO_*x*_/SiO_*y*_) junctions, which is related to some kinds of defects produced during the growth process, as shown in the FTIR spectra and listed in Table 5.

**Table 5:** **Defect types linked**[19-24]**with the peak position**

**Defect type**	**Peak position (nm)**					
	**As grown**					
	**1,150/1,020°C**	**1,150/900°C**	**1,020/1,150°C**	**1,020/900°C**	**900/1,150°C**	**900/1,020°C**
WOB	-	-	-	408	406	414
NOV defect	449	438	432	-	-	-
E'δ defect	532	534	-	559	542	514
NBOHC	648	622	667	674	672	644
None identified	805	782	795	789	770	780

Peak position obtained by deconvolution from PL spectra of the (SiO_*x*_/SiO_*y*_) junctions as function of the Tg.

Figures 6b and 7b show the PL spectra of the SiO_*x*_ films and (SiO_*x*_/SiO_*y*_) junctions with thermal annealing, respectively. It is observed that when the thermal annealing is applied to the samples, a restructuring occurs (as shown in the FTIR spectra) because the temperature causes a phase separation and growth of Si-nc's. For the case of the SiO_*x*_ films, the defect-related PL is almost eliminated and the near-infrared PL intensity (band B) is a little enhanced after annealing, i.e., the PL is dominated by the Si-nc's embedded in the SiO_*x*_ matrix. For the case of the (SiO_*x*_/SiO_*y*_) junctions, what is most interesting is that they emit not only in the orange-near infrared range (band B) but also in the violet-blue range (band A), and the green-yellow band disappears. In this case, the PL intensity of band A slightly declines with respect to the PL intensity of the (SiO_*x*_/SiO_*y*_) junctions as-grown. So the dominant radiative defects have changed from E'δ or NBOHC defects to Si-O species (i.e., WOB and NOV defects). With respect to the PL intensity of band B, this is greatly enhanced after thermal annealing. It is reasonable to consider that for the (SiO_*x*_/SiO_*y*_) junctions, the two mechanisms are not contradictory but run parallel in the samples. In summary, two PL bands, band A and band B, have been observed after thermal annealing. According to the PL spectra, the former band A is ascribed to Si-O-related species [19,20,24], and band B is ascribed to the Si-nc's embedded in the SiO_*x*_ matrix [9]. Annealing seems to advance the formation of crystalline silicon (c-Si) as well as the formation of (defect) structures responsible for PL emission. The difference in behavior of the PL intensity before and after annealing between the SiO_*x*_ films and junctions is interesting; this difference could be due to the fact that the single layers have a combination of defects and formed Si-nc's which generates intense PL. When annealing SiO_*x*_ films, the defects are desorbed and generated, leading to the decrease in PL intensity. In double layers, there are several factors, one is the double annealing procedure and the other is the order of layers, which have a very strong impact on optical properties, where the defects of the first layer are preserved due to the growth of the second layer. These layers have a higher concentration of defects and excess silicon; when annealing (SiO_*x*_/SiO_*y*_) junctions, some defects are desorbed or modified, mainly on the second layer, where the PL is improved in the red. According to the analysis of the compositional and optical properties of the SiO_*x*_ films and (SiO_*x*_/SiO_*y*_) junctions, we can correlate the evolution of the stoichiometry (obtained by FTIR and XEDS) with the reduction of the Eg (obtained by transmittance), where the oxygen content decreased along with the increase in growth temperature and in turn with the reduction of the Eg. So, we may assume that the Si excess increases as the growth temperature increases.

## Conclusions

In the present work, SiO_*x*_ films and (SiO_*x*_/SiO_*y*_) junctions were obtained by HFCVD technique with different growth temperatures; their compositional and optical properties were studied as-grown and after further annealing. For the case of the SiO_*x*_ films, as-grown, the absorption and emission are correlated with quantum effects in silicon nanoparticles and also associated with defects. For the case of the (SiO_*x*_/SiO_*y*_) junctions, as-grown, only the second mechanism of light emission was considered, which is related to some kinds of defects produced during the growth process. After thermal annealing, the PL spectra of the SiO_*x*_ films only show band B (740 nm), and the PL spectra of the (SiO_*x*_/SiO_*y*_) junctions show bands A (375 to 450 nm) and B (740 nm), where the PL band A is ascribed to Si-O-related species, and the PL band B to the quantum size effect of the Si-nc's embedded in the SiO_*x*_ matrix. Vibration bands related to Si-H was observed in SiO_*x*_ films and (SiO_*x*_/SiO_*y*_) junctions before annealing (as shown in the FTIR spectra), which disappeared after thermal annealing. The behavior of the change in intensity and shift of the FTIR and PL spectra is ascribed to changes of phase in this material, associated with several defects and Si-np's. The (SiO_*x*_/SiO_*y*_) junctions showed an interesting behavior due to the double annealing procedure and ‘order’ of layers, which have a very strong impact on optical and compositional properties. This opens the possibility of tuning the spectra range for a specific application.

## Competing interests

The authors declare that they have no competing interests.

## Authors' contributions

DEVV conducted the SiO_*x*_ growth. DEVV and JALL carried out the FTIR and PL measurements and drafted the manuscript. JCL coordinated the FTIR study. GGS coordinated and participated in the growth of the SiO_*x*_ films. ABL conducted the thermal annealing. NDET conducted the UV measurements. JALL provided the idea and supervised the study. All authors read and approved the final manuscript.

## Authors' information

DEVV is currently a PhD student in the Science Institute - Center of Investigation in Semiconductors Devices (IC-CIDS) from Autonomous University of Puebla, Mexico. She started to work on the growth and characterization of non-stoichiometric silicon oxide obtained by HFCVD. Her research interests include experiments and structural, optical and electrical characterization of SiO_*x*_ films and MOS structures. JALL is currently a researcher and professor in the Science Institute - Center of Investigation in Semiconductors Devices (IC-CIDS) from Autonomous University of Puebla, Mexico. He started to work on structural, electrical and optical characterization of materials and MOS structures. His research interest is the physics and technology of materials nanostructures and silicon devices. Moreover, his research interests are, too, the nanotechnology, material characterization, and optoelectronic devices such as sensors, LEDs, and solar cells. GGS received his PhD in the Electronic and Solid State Department from the Center of Research and Advanced Studies, National Polytechnic Institute, Mexico City in 2003. He started to work on the growth and characterization of non-stoichiometric silicon oxide. His current research interests include metallic oxides obtained by the HFCVD technique, GaN obtained by the metal organic CVD technique and porous silicon gas sensor devices.
